# Pathophysiological aspects of nephropathy caused by non-steroidal anti-inflammatory drugs

**DOI:** 10.1590/2175-8239-JBN-2018-0107

**Published:** 2018-09-21

**Authors:** Guillherme Nobre Cavalcanti Lucas, Ana Carla Carneiro Leitão, Renan Lima Alencar, Rosa Malena Fagundes Xavier, Elizabeth De Francesco Daher, Geraldo Bezerra da Silva

**Affiliations:** 1 Universidade de Fortaleza, Programa de Pós-Graduação em Saúde Coletiva, Centro de Ciências da Saúde, Fortaleza, CE, Brasil.; 2 Universidade do Estado da Bahia, Curso de Farmácia, Salvador, BA, Brasil.; 3 Universidade Federal da Bahia, Instituto de Saúde Coletiva, Salvador, BA, Brasil.; 4 Universidade Federal do Ceará, Faculdade de Medicina, Departamento de Medicina Clínica, Fortaleza, CE, Brasil.

**Keywords:** Anti-Inflammatory Agents, Drug-Related Side Effects and Adverse Reactions, Toxicity, Physiopathology, Review, Anti-Inflamatórios, Efeitos Colaterais e Reações Adversas Relacionados a Medicamentos, Toxicidade, Fisiopatologia, Revisão

## Abstract

Non-steroidal anti-inflammatory drugs (NSAIDs) are commonly used medications associated with nephrotoxicity, especially when used chronically. Factors such as advanced age and comorbidities, which in themselves already lead to a decrease in glomerular filtration rate, increase the risk of NSAID-related nephrotoxicity. The main mechanism of NSAID action is cyclooxygenase (COX) enzyme inhibition, interfering on arachidonic acid conversion into E2 prostaglandins E2, prostacyclins and thromboxanes. Within the kidneys, prostaglandins act as vasodilators, increasing renal perfusion. This vasodilatation is a counter regulation of mechanisms, such as the renin-angiotensin-aldosterone system works and that of the sympathetic nervous system, culminating with compensation to ensure adequate flow to the organ. NSAIDs inhibit this mechanism and can lead to acute kidney injury (AKI). High doses of NSAIDs have been implicated as causes of AKI, especially in the elderly. The main form of AKI by NSAIDs is hemodynamically mediated. The second form of NSAID-induced AKI is acute interstitial nephritis, which may manifest as nephrotic proteinuria. Long-term NSAID use can lead to chronic kidney disease (CKD). In patients without renal diseases, young and without comorbidities, NSAIDs are not greatly harmful. However, because of its dose-dependent effect, caution should be exercised in chronic use, since it increases the risk of developing nephrotoxicity.

## Resumo

Os anti-inflamatórios não esteroidais (AINEs) são medicamentos comumente utilizados, associados à nefrotoxicidade, sobretudo quando utilizados cronicamente. Fatores como idade avançada e comorbidades, que por si só já levam à diminuição da taxa de filtração glomerular, aumentam o risco de nefrotoxicidade dos AINEs. O principal mecanismo de ação dos AINEs é a inibição da enzima ciclooxigenase (COX), interferindo na conversão do ácido araquidônico em prostaglandinas E2, prostaciclinas e tromboxanos. Nos rins, as prostaglandinas atuam como vasodilatadoras, aumentando a perfusão renal. Essa vasodilatação atua como uma contrarregulação de mecanismos, como a atuação do sistema renina-angiotensina-aldosterona e do sistema nervoso simpático, culminando com uma compensação para assegurar o fluxo adequado ao órgão. O uso de AINEs inibe esse mecanismo, podendo causar lesão renal aguda (LRA). Altas doses de AINEs têm sido implicadas como causas de LRA, especialmente em idosos. A principal forma de LRA por AINEs é a hemodinamicamente mediada. A segunda forma de apresentação da LRA induzida por AINES é a nefrite intersticial aguda, que pode se manifestar com proteinúria nefrótica. O uso de AINEs em longo prazo pode ocasionar doença renal crônica (DRC). Nos pacientes sem doenças renais, jovens e sem comorbidades, os AINEs não apresentam grandes malefícios. Entretanto, por seu efeito dose-dependente, deve-se ter grande cautela no uso crônico, por aumentar risco de desenvolver nefrotoxicidade.

## INTRODUCTION

Non-steroidal anti-inflammatory drugs (NSAIDs), often prescribed in medical practice as analgesic, antipyretic and anti-inflammatory agent, are among the most widely used drug classes worldwide. Recent studies point to NSAIDs as the most effective drugs, for example, for the treatment of pain associated with renal calculi, being better even than opioids.^1^ The main consumers of this group of drugs are individuals afflicted by chronic pain, usually associated with rheumatologic diseases, including rheumatoid arthritis, osteoarthritis and other musculoskeletal disorders.^2^^,^ 3 ^,^ 4 The pharmacological action of NSAIDs depends on the dose and duration of use, which predisposes the involvement of specific organs, and the second one most affected are the kidney. Therefore, it is one of the drugs that, if used in the long term, increases morbidity, especially for the elderly, since they use several other medications (antihypertensives, antidepressants, anticoagulants) that may cause interactions. These patients are likely to develop kidney injury, which may be transitory or not. However, those exposed by a prolonged use of drugs are those with chronic kidney disease, with a 3 to 4-fold increase in risks of adverse effects.^5^

In addition to renal complications, NSAIDs can cause gastrointestinal (gastric perforation and ulceration), hepatic (cirrhosis), cardiovascular and platelet (thrombotic events) alterations, requiring caution and proper indications in its prescription.^6^

## MECHANISM OF ACTION OF NSAIDS

The main mechanism of NSAID action is the cyclooxygenase (COX) enzyme inhibition, both centrally and peripherally, thus interfering with the conversion of arachidonic acid into E2 prostaglandins, prostacyclins and thromboxanes. Prostaglandins have a vasodilatation effect, which is extremely important for preglomerular resistance maintenance, maintaining glomerular filtration rate and preserving renal blood flow.^7^

Enzymes related to the action of NSAIDs can be divided into COX-1 and COX-2, acting in different regions. COX-1 is the one that occurs in most cells, even fetal and amniotic fluid, and participates in physiological effects, such as regulatory and protective effects. COX-2 is activated by inflammation and pro-inflammatory cytokines.^8^

Based on the classification of these enzymes, NSAIDs can be classified into non-target NSAIDs (ketoprofen, aspirin, naproxen, flunixin, meglumine and others), COX-2 preferential inhibitors (meloxican, etodolac, nimesulide) and highly selective COX-2 inhibitors (coxib). Most of the side effects are related to COX-1 inhibition, due to its action in several systems associated with cell cleansing. In the kidneys, they are in greater quantities acting in glomerular filtration maintenance. Therefore, studies indicate that individuals with previously compromised renal function are the most affected by the time-dependent use of non-selective NSAIDs. The action of COX-2 is associated with water and electrolytic maintenance in the renal environment, which worsens its effects under dehydration, low renal perfusion or previously existing renal damage.^7^

## PHYSIOPATHOLOGY OF NSAID-RELATED KIDNEY INJURY

The kidneys are important organs for the excretory function of the body because they receive about 25% of all cardiac output.^1^ In order to adequately perform their filtration function, these organs have regulatory mechanisms, such as prostaglandin synthesis, which will maintain glomerular filtration rate (GFR) and renal homeostasis.^9^

NSAIDs inhibit the cascade of arachidonic acid, selectively or not, causing a nonpermissive effect on the formation of prostaglandins.^10^ In the kidneys, prostaglandins - mainly prostacyclins, PGE2, PGD2 - will act as vasodilators in the afferent arteriole, increasing renal perfusion, with distribution of the cortex flow to the nephrons in the renal medullary region. This vasodilatation acts as a negative feedback on the mechanisms, such as the performance of the renin-angiotensin-aldosterone system and the sympathetic nervous system, culminating with compensation to ensure adequate flow to the organ. NSAIDs inhibit this mechanism and may result in acute vasoconstriction and spinal cord ischemia, which can lead to acute renal injury.^2^^,^ 9 ^,^ 10

In addition to the vasodilatation action, through the stimulation of tubular receptors PGE2 will inhibit the transport of sodium and chloride in the ascending loop of Henle and in the collecting ducts, by means of stimulating of the EP1 receptor, leading to natriuresis. In addition, PGE2 exerts an antagonistic action on the antidiuretic hormone (ADH) receptors, also promoting diuresis.^9^^,^ 11 ^,^ 12 NSAIDs may also cause higher sodium and water retention by inhibiting PGE2 production, leading to the formation of edema, which is often subclinical.^8^ Clinical trials comparing different NSAIDs show the development of hypertension, especially when using high doses and for a prolonged time, with ibuprofen being more involved.^13^

In addition to its actions in the kidneys, prostaglandins perform several functions related to homeostasis, such as protection of the gastrointestinal mucosa, platelet activation, inflammation, bronchodilation, and others.^2^

**Figure 1:**
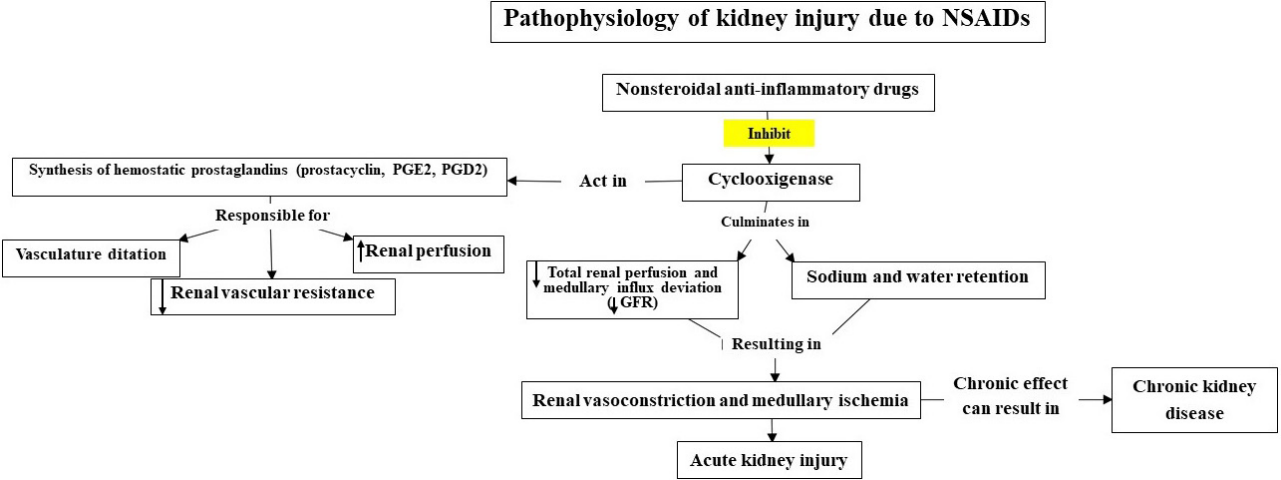
NSAID-induced kidney injury pathophysiology

Figure 1 shows the pathophysiology of renal injury induced by NSAIDs.

## RISK FACTORS

Kidney damage caused by the use of NSAIDs is not common, especially when it comes to individuals who are previously healthy and who do not use abusive or high doses of these drugs. Some factors, such as advanced age and comorbidities, which in themselves already lead to a decrease in GFR, increase the risk of NSAID-related nephrotoxicity, contributing to the development of side effects. One of the risk factors is systemic arterial hypertension, which causes an even higher activation of the renin-angiotensin-aldosterone system (RAAS) and the sympathetic nervous system, leading to vasoconstriction; and the inhibition of prostaglandin synthesis causes the loss of the compensatory mechanism of renal vasodilation.^14^

The same applies to comorbidities that lead to a decrease in effective arterial volume, such as nephrotic syndrome with a high level of proteinuria, liver cirrhosis, especially in those with ascites, heart failure and lupus nephritis. Patients with these conditions using NSAIDs have an inhibition of the kidney-compensation mechanism, as it happens in hypertensive patients, which contributes to renal damage.^2^^,^ 3 ^,^ 8 ^,^ 14

## NSAIDs AND HYDROELETROLYTIC DISORDERS

As already explained above, prostaglandins (PGs) play an important role in maintaining renal activity. Renal vasodilatation induced by PGs is critical for maintenance of adequate kidney perfusion by PGE2 and PGI2. The main hydroelectrolytic and acid-basic changes caused by this class of drugs are sodium retention (causing edema and hypertension), hyperkalemia and metabolic acidosis due to the lower activity of COX-1 and COX-2.^4^^,^ 15

The inhibition of prostaglandin-mediated vasodilatation (PGE-2) prevents adequate renal perfusion. In circulating arterial volume dysregulation situations, in which there is higher RAAS stimulation, there is a high production of prostaglandins (PGE-2, PGI-2) by the afferent arteriole endothelium. These prostaglandins are a self-regulating mechanism in cases of decreased renal perfusion, such as heart failure, hypovolemia, and as compensatory vasodilatation of the afferent arteriole mediated by PGE2, that occurs in response to norepinephrine or angiotensin II action.^2^^,^ 6

There is an increased synthesis in glomerular disease, renal failure (GFR <60mL/minute/1.73m^2^), hyperkalemia, heart failure, cirrhosis and hypovolemic shock, states of circulating arterial volume reduction, which implies a greater stimulus for PG synthesis. If production is reduced, it is only natural for plasma retention to occur due to afferent arteriolar vasoconstriction, leading to hydroelectrolytic and acid-base disturbances, especially water retention (resulting from increased sodium reabsorption) and hyperkalemia. Thus, patients with such conditions associated with the use of NSAIDs are more vulnerable to developing nephrotoxicity.^2^^,^ 14 ^,^ 16

NSAIDs may reduce response to diuretics by about 20%, especially loop diuretics, and such effect may be more commonly expressed in chronic sodium retainers, such as those with congestive heart failure.

Aldosterone is able to increase potassium excretion. Since prostaglandin PGI2 stimulates renal juxtaglomerular cells to release renin and, consequently, aldosterone (a state of hypoaldosteronism), inhibition of the production of this molecule by NSAIDs can cause less distal tubular flow, resulting in hyperkalemia, as well as metabolic acidosis. Recent evidence from case-control and retrospective studies suggest that there is no correlation between a higher incidence of hyperkalemia with selective COX-2 inhibitors (the coxibs), as already described in clinical trials of the 1980s, and thus the incidence of hypokalemia with any class of NSAIDs.^4^^,^ 9 ^,^ 14 ^,^ 17

COX-1 acts primarily in the control of renal GFR, while COX-2 plays a role in sodium and water excretion. Blocking both enzymes prevents PGE2 production. This enzyme regulates the reabsorption of sodium and water in the renal tubules (diuretic and natriuretic effect), besides optimizing blood perfusion to the renal medulla, which contributes to this effect. PGE2 is considered a tubular PG, while PGI2 is vascular. However, in physiological situations, such enzymes are not primary components of the hydroelectrolytic homeostasis generated in the kidneys, since the baseline production rate of prostaglandins is relatively low.^2^^,^ 4 ^,^ 11 ^,^ 12 ^,^ 15 ^,^ 17 It has been suggested that the acute sodium retention caused by NSAIDs in healthy elderly is mediated by COX-2 inhibition, while the sudden decrease in GFR is due to COX-1 inhibition.^4^

Thus, NSAIDs with little or no effect on COX-2, such as aspirin, rarely cause obvious sodium retention and hypertension.^18^ In the SUCCESS VI and VII trials, there was a significant increase in the number of patients with high blood pressure in an elderly population (> 65 years), using coxibs, especially rofecoxib.^19^^,^ 20

Hyponatremia induced by NSAIDs is possibly correlated with the lower release of PGE2 and PGI2, which antagonize the action of vasopressin in water absorption in collecting tubules, although there is a negative feedback NSAID mechanism of sodium retention by prostanoid inhibition, which is the main cause of the overfilling effect due to arterial hypertension and edema. It is suggested that sodium retention by NSAIDs is mediated by COX-2.^12^^,^ 15 ^,^ 17

Several statements regarding the renal effects of NSAIDs have been proven in experimental animal studies. In rats with volume depletion by oral administration of furosemide, the use of indomethacin (non-selective inhibitor) and flosulide (selective COX-2 inhibitor) caused renal hypoperfusion and decreased GFR, in addition to the expected decrease in urinary prostaglandins, indicating that NSAIDs and non-COX-2 inhibitors alter renal function in hypovolemic animals.^21^ Another study demonstrated a correlation between the effects of both classes (COX-1 and COX-2 inhibitors) causing low rennin levels in cats. Burukoglu et al.^10^ ratified these results with the use of meloxican in rats. Hypomagnesemia and hypophosphatemia can be established within one to two days after an episode of excessive NSAIDs intake.^22^

## NSAID AND ACUTE RENAL INJURY

Acute kidney injury comprises a syndrome characterized by abrupt GFR reduction, leading to retention of urea, creatinine, and other nitrogen waste substances that are normally cleared by the kidneys. This condition is clinically defined when patients increase creatinine levels within a few days (or it is 1.5 times higher in relation to a recent or presumed outcome) or who develop oliguria/anuria, justifying high morbidity and mortality in the emergency room. Virtually all NSAIDs can be associated with AKI.^23^^,^ 24 Arterial blood gas sampling is often the essential admission test if the patient has uremic syndrome signs, such as progressive decline in mental status and seizures, or signs of acute kidney injury (such as oliguria), leading to metabolic acidosis caused by excessive intake of NSAIDs.^22^ High doses of NSAIDs have been implicated as cause for AKI, especially in the elderly.^12^ However, NSAID-mediated AKI is a rare condition.^24^

The main form of acute kidney injury caused by NSAIDs is hemodynamically mediated. In contrast, in situations of chronic kidney disease, heart failure, liver failure, hypovolemic shock and other conditions that reduce circulating arterial volume, the secretion of these hormones increases in order to preserve renal perfusion and GFR. The breakdown of this process by NSAIDs results in reduction of intramedullary renal perfusion and ischemia, increasing the risk of acute tubular necrosis (ATN). Some evidence suggests a lower nephrotoxic potential in low dose non-selective COX drugs, such as ASA and ibuprofen, in comparison to selective COX-2 agents.^25^^,^ 26

The second form of presentation of NSAID-induced AKI is acute interstitial nephritis (AIN) with nephrotic syndrome. Nephrotic proteinuria is reported in about 80% of patients, more often associated with phenoprofen, naproxen and ibuprofen.^27^ AIN may also happen without nephrotic syndrome. The exact mechanisms of the pathophysiology of NIAs triggered by NSAIDs are not yet known and have been attributed to a delayed hypersensitivity reaction, with the main factors that point to this mechanism being: need for prolonged exposure to NSAIDs, low frequency of the classic signs of hypersensitivity and interstitial infiltrate with predominance of T-lymphocytes.^27^ It is also described a deviation of the arachidonic acid metabolism for the formation of leukotrienes and derivatives, activating T-lymphocytes, resulting in interstitial infiltration, leading to the onset of minimal lesion disease (MLD), with nephrotic syndrome (edema, oliguria, proteinuria) a few days after treatment onset. Renal function is usually restored after drug interruption.^4^

Therapeutic doses of NSAIDs in susceptible patients can cause acute renal injury. The explanation for this comes from the same mechanism previously explained: biosynthesis inhibition of the prostanoids involved in the maintenance of renal blood flow, specifically PGE2 and PGI2. The risk is higher in neonates and the elderly, as well as in patients with some cardiovascular, liver, kidney, or chronic disease, or with reduced circulating blood volume, such as patients using NSAIDs combined with diuretics and RAAS inhibitors.^5^^,^ 12 Dreischulte et al.,^28^ in a case-control study with almost 80,000 users on prolonged use of NSAIDs associated with diuretics and/or angiotensin converting enzyme (ACE) inhibitors, showed a strong relationship. It was reported that combined therapy compared to NSAID monotherapy was responsible for an absolute increase in the risk of community-acquired AKI in one year of use, despite the high rate of AKI caused by the exclusive use of anti-inflammatory drugs, being the main drug class causing renal dysfunction.^28^

## NSAID-INDUCED CHRONIC RENAL DISEASE

There are not many studies yet showing the long-term effects of NSAIDs on the development of chronic kidney disease (CKD). However, it has been shown that daily use for more than one year increases the risk of developing CKD.^14^

There may be progression in patients who do not discontinue NSAIDs when they develop acute interstitial nephritis and interstitial fibrosis.^5^ A recent study in the elderly population shows that regardless of the class of this medication, whether selective or not, both high doses and longer half-lives significantly increase the risk of CKD development.^11^^,^ 29

The main effects of NSAIDs on renal function are summarized in Table 1.

**Table 1:** Main NSAID effects on kidney function

	Mechanisms	Risk factors	Prevention/Treatment
Water and electrolyte disorders	PGE2 and PG12-induced kidney vasodilatation inhibition; RAAS activation	NSAID use (most common nephrotoxic effects	Discontinue NSAID use
-Sodium retention			
-Hyperkalemia			
-Hyponatremia			
-Metabolic acidosis			
-Lower response to diuretics (especially loop diuretics)			
Acute kidney injury	Hemodynamic alterations/Kidney perfusion reduction	Liver diseases; Kidney diseases; Heart failure; Dehydration; advanced age	Avoid in high-risk patients (patients with comorbidities); Discontinue NSAID
Acute interstitial nephritis	Hypersensitivity reaction	Prolonged NSAID exposure; some specific NSAIDs (Phenoprofen, Naproxen, Ibuprofen)	Discontinue NSAID use
Chronic kidney disease	Hemodynamic alterations	Chronic use of NSAIDS	Avoid use in high-risk patients (those with comorbidities and advanced age); Discontinue NSAID use
Papillary necrosis	Direct toxicity	Phenacetine abuse; Aspirin and acetaminophen combination	Discontinue NSAID use and avoid chronic use of analgesics

*PGE2: prostaglandins; PGI2: prostacyclins; RAAS: Renin-angiotensin-aldosterone system; NSAIDs: Non-steroidal anti-inflammatory drugs. Adapted from: Melgaço *et al*.^14^

## CONCLUSION

NSAIDs do not present great harm to patients without renal diseases, young and without comorbidities. However, because of its dose-dependent effect, great caution should be exercised in chronic use of these agents, because it increases the chances of developing some toxicity and morbidity. NSAID agents, selective and non-selective, directly interfere with renal function due to prostaglandin inhibition, and can cause mild and transient disorders to chronic kidney disease. Therefore, the indication of this class of drugs should be well evaluated, always verifying the risk-benefit, besides taking into consideration the patient in question and the potential effects caused by its use.
